# HLA–DRB1 Amino Acid Positions 11/13, 71, and 74 Are Associated With Inflammation Level, Disease Activity, and the Health Assessment Questionnaire Score in Patients With Inflammatory Polyarthritis

**DOI:** 10.1002/art.39780

**Published:** 2016-10-27

**Authors:** Stephanie F. Ling, Sebastien Viatte, Mark Lunt, Alper M. Van Sijl, Lucia Silva‐Fernandez, Deborah P. M. Symmons, Adam Young, Alexander J. Macgregor, Anne Barton

**Affiliations:** ^1^ University of ManchesterManchesterUK; ^2^ University of Manchester, Manchester, UK, and Jan van Breemen Research Institute ReadeAmsterdamThe Netherlands; ^3^ University of Manchester, Manchester, UK, and Complexo Hospitalario Universitario de FerrolA CoruñaSpain; ^4^ University of Manchester and Central Manchester University Hospitals NHS Foundation TrustManchesterUK; ^5^ St. Albans City Hospital, St. Albans, UK, and University of HertfordshireHatfieldUK; ^6^ University of East AngliaNorwichUK

## Abstract

**Objective:**

Rheumatoid arthritis (RA) susceptibility HLA–DRB1 haplotypes based on amino acid positions 11/13, 71, and 74 predict radiographic damage. The mechanism of action is unknown, but it may be mediated by inflammation. We undertook this study to systematically investigate the effect of these amino acids on nonradiographic measures of disease activity/outcomes.

**Methods:**

We tested the association of RA susceptibility HLA–DRB1 amino acids with the C‐reactive protein (CRP) level, the tender joint count (TJC), the swollen joint count (SJC), the Disease Activity Score in 28 joints (DAS28), and the Health Assessment Questionnaire (HAQ) score in the Norfolk Arthritis Register (NOAR) and Early Rheumatoid Arthritis Study (ERAS) cohorts. Longitudinal modeling of disease activity/outcomes was performed using generalized linear latent and mixed models. Mediation analysis was performed using directed acyclic graphs to investigate the paths from genetic factors to outcome.

**Results:**

A total of 2,158 patients were available for analysis in the NOAR cohort. Valine at position 11 showed the strongest association with the CRP level (*P* = 2.21 × 10^−6^), the SJC (*P* = 7.51 × 10^−6^), and the DAS28 (*P* = 0.002); it was marginally associated with the HAQ score (*P* = 0.044) but not with the TJC. The same amino acid and haplotype risk hierarchy observed for susceptibility and radiographic severity was observed for the CRP level and nonradiographic measures of disease activity/outcome, apart from the TJC. The results were replicated in the ERAS cohort. The effect of valine at position 11 on the SJC was mainly mediated by anti–citrullinated protein antibody status, the effect of which was mainly mediated by inflammation; however, the effect of valine at position 11 was also independent of the CRP level (*P* = 1.6 × 10^−4^).

**Conclusion:**

Genetic markers of RA susceptibility located within HLA–DRB1 determine the levels of clinical and systemic inflammation independently, and also determine all objective measures of disease activity and outcome.

The shared epitope (SE) hypothesis postulates that HLA–DRB1 alleles sharing a similar amino acid motif at positions 70–74 confer susceptibility to rheumatoid arthritis (RA) 1. However, it has recently been demonstrated that a model including amino acids at positions 11 or 13 (both are in tight linkage disequilibrium [LD]), 71, and 74 better explains the association of HLA–DRB1 with RA susceptibility 2.

Amino acid combinations from these 3 positions (which are in partial LD with one another) form 16 haplotypes hierarchically classified with regard to their effect on RA susceptibility (from conferring risk to conferring protection) 2. They are independently associated with radiographic damage and mortality, with an identical risk hierarchy between susceptibility and radiographic severity, demonstrating that position 11/13 (outside the SE) also contributes to progression and outcome 3. Valine at position 11 has been reported to influence radiographic progression in RA patients in 2 further independent cohorts 4, 5.

However, it is unclear by what mechanism the 16‐haplotype model results in radiographic damage. Previous work has suggested that high levels of C‐reactive protein (CRP) correlate with radiographic outcomes 6; however, it has also been shown that RA can progress in the absence of detectable systemic inflammation 7, 8. Acute‐phase markers are imperfectly correlated with objective evidence of synovitis, and therefore, the Disease Activity Score in 28 joints (DAS28) 9, swollen joint count (SJC), or tender joint count (TJC) may be better markers of chronic inflammation. It has been shown that clinical (e.g., the SJC) rather than laboratory (or systemic) (i.e., the CRP level or erythrocyte sedimentation rate [ESR]) measures of inflammation are associated with radiographic progression and mortality 8, but the correlation of HLA–DRB1 markers with any measures of inflammation (clinical or laboratory) has not been studied yet. We hypothesize that the effect of HLA–DRB1 haplotypes on outcome is mediated via inflammation.

Therefore, we aimed to first examine underlying pathways driving radiographic damage by testing genetic associations of the 16‐haplotype model with the CRP level. Second, we examined genetic associations with other nonradiographic measures of disease outcome and activity (including the DAS28 and its subcomponents [TJC, SJC, and CRP level]) and with disability using the Health Assessment Questionnaire (HAQ) 10. Third, we performed mediation analysis using directed acyclic graphs to investigate the relative contribution of different path variables in the disease process (CRP level, anti–citrullinated protein antibody [ACPA] status, and the like).

## PATIENTS AND METHODS

### Patients and cohorts

Two prospective cohorts of patients with longitudinal outcome data were studied. These were the Norfolk Arthritis Register (NOAR) 11 and the Early Rheumatoid Arthritis Study (ERAS) 12 cohorts. NOAR is a primary care–based inception cohort of 2,158 patients recruited since 1989 (recruitment is still ongoing) presenting with recent‐onset inflammatory polyarthritis, defined as having ≥2 swollen joints for >4 weeks. Patients recruited between 1989 and 1994 remained under long‐term follow‐up (up to 20 years’ duration), with the exception of 1) patients who did not satisfy the American College of Rheumatology (ACR) 1987 revised criteria for RA 13 at 5 years of follow‐up and who were diagnosed by a consultant as having disease other than RA, undifferentiated inflammatory polyarthritis, psoriatic arthritis, or postviral arthritis to explain their symptoms and 2) patients with spontaneous long‐term remission of their disease, defined as having no inflamed joints at the third or fifth annual follow‐up visits and not taking disease‐modifying antirheumatic drugs (DMARDs)/steroids.

Consequently, patients recruited between 1989 and 1994 were more likely to be followed up after year 5 if they had high disease severity. This can reduce study power compared to a study design without these exclusion criteria, but it will not affect the effect size of genetic markers tested in the present study, as the relative change in disease activity/severity measures between carriers and noncarriers of specific genetic markers will be unaffected. Censoring the study at year 5 or excluding all patients recruited between 1989 and 1994 would have a much more drastic impact on power, so the inclusion of such patients is appropriate.

All patients, including RA patients, were called inflammatory polyarthritis patients. Most inflammatory polyarthritis patients will satisfy the ACR 1987 revised criteria for RA during follow‐up. CRP level was measured at 0, 5, 10, and 15 years; consequently, the DAS28 using the CRP level was only calculated at these time points as well. The TJC and SJC were done at 0–3, 5, 10, 15, and 20 years. HAQ scores were obtained at 0–5, 7–8, 10, 12, 15, 18, and 20 years.

ERAS is a tertiary care–based cohort of 329 adults clinically diagnosed as having RA who were recruited consecutively from rheumatology outpatient clinics, had a symptom duration of <2 years, and had no use of second‐line medication. Patients who did not fulfill the ACR 1987 revised criteria for RA 13 continued to be followed up and were included for analysis. Patients were excluded if the diagnosis changed from RA (e.g., early RA evolving to classical systemic lupus erythematosus). The TJC, SJC, ESR, DAS28 using the ESR, and HAQ score were recorded every year, with a maximum follow‐up of 15 years. The NOAR and ERAS patients in the present study are the same as those studied in our previous work 3.

### Genotyping

Genotyping was carried out as previously described 3, 14. Sixteen possible haplotypes exist, based on the combinations of amino acids at HLA–DRB1 positions 11, 71, and 74 (2) (Table 1). Only results from haplotypes with a frequency of >5% are reported in NOAR, and only results from haplotypes with a frequency of >10% are reported in ERAS, although analysis was carried out on all 16 possible haplotypes in both univariate and multivariate models. A more stringent frequency cutoff was required in ERAS due to the smaller sample size.

**Table 1:** Derivations of the 16‐haplotype classification, from amino acids at positions 11, 71, and 74

Position 11	Position 71	Position 74	Haplotype name	Classical HLA–DRB1 alleles
Valine	Lysine	Alanine	VKA^a^	*04:01
Valine	Arginine	Alanine	VRA^a^	*04:08, *04:05, *04:04, *10:01
Leucine	Arginine	Alanine	LRA^a^	*01:02, *01:01
Proline	Arginine	Alanine	PRA	*16:01
Valine	Arginine	Glutamic acid	VRE	*04:03, *04:07
Aspartic acid	Arginine	Glutamic acid	DRE	*09:01
Valine	Glutamic acid	Alanine	VEA	*04:02
Serine	Lysine	Alanine	SKA	*13:03
Proline	Alanine	Alanine	PAA^a^	*15:01, *15:02
Glycine	Arginine	Glycine	GRQ^a^	*07:01
Serine	Arginine	Alanine	SRA^a^	*11:01, *11:04, *12:01
Serine	Arginine	Glutamic acid	SRE	*14:01
Leucine	Glutamic acid	Alanine	LEA	*01:03
Serine	Arginine	Leucine	SRL	*08:01, *08:04
Serine	Lysine	Arginine	SKR^a^	*03:01
Serine	Glutamic acid	Alanine	SEA^a^	*11:02, *11:03, *13:01, *13:02

a High‐frequency haplotype (≥5% study population frequency). The classification system is derived from Raychaudhuri et al (2).

### Statistical analysis

#### Statistical modeling

Since disease outcome is variable over time and both NOAR and ERAS are prospective cohorts of patients with multiple records over time (data available for up to 20 years in NOAR and for up to 15 years in ERAS), we performed a longitudinal modeling of disease activity/outcome. The DAS28, TJC, SJC, CRP level/ESR, and HAQ score were modeled using Stata software version 13.1 (StataCorp) as longitudinal continuous non‐normally distributed outcome variables using a generalized linear latent and mixed model (GLLAMM) 15 with discrete random effects and a varying number of latent classes, depending on the distribution of each variable. An additive model of association was created for all analyses by creating a numerical variable (0, 1, or 2) for the number of alleles carried by each patient. An allele is defined either as a specific amino acid at a specific position or as a haplotype. A haplotype is defined as a combination of amino acids coded by DNA positions inherited independently of one another. The statistical effects are reported for one copy of every allele.

#### Covariates

When performing longitudinal modeling of disease outcome, disease duration and patient age are known factors associated with disease outcome in RA 16, 17. We therefore adjusted every model for the effect of age at symptom onset and duration of symptoms at each time point. Orthogonal polynomials of these variables were added to the model in order to take the nonlinear relationship between these covariates and disease outcome into consideration; orthogonal polynomials were used due to strong correlation between the terms of regular polynomials. The statistics Akaike's information criterion and Bayesian information criterion were used to determine model parameters (e.g., the power of polynomials) prior to analysis to ensure best model fit. When subanalysis was carried out to adjust for treatment, we incorporated a time‐dependent categorical treatment variable (presence or absence of treatment with a DMARD at every time point studied). The inclusion of further covariates (e.g., ACPA status) is explained below in the section on mediation analysis.

#### Data censoring

All time points with available data on the following variables studied in NOAR were included: TJC, SJC, and CRP level. Data were censored at 15 years for the DAS28 and at 5 years for the HAQ score. Data censoring was carried out because previous work by our group has shown that radiographic damage at different time points is strongly correlated, but the variance in damage peaks during initial follow‐up and decreases from there onward 17. Consequently, longitudinal modeling must be censored at a time point that depends on the outcome being studied. A sensitivity analysis was performed to determine the above censoring cutoffs.

#### Effect sizes

The effect size of a GLLAMM for the DAS28, TJC, SJC, and HAQ score is a β coefficient, representing the change in the dependent variable for every additional copy of the amino acid or haplotype tested, expressed in the same units. CRP level and ESR required log transformation for analysis due to their distributions, so results are reported as percentage change in CRP level/ESR, following exponentiation of the β coefficient. Effect sizes are reported per copy of every allele, compared to a reference group. Effect sizes would be larger if expressed for homozygote carriers and if the reference group would be a protective genotype. As an example, the average difference in the DAS28 between a VKA homozygote carrier (higher risk haplotype) (Table 1) and an SEA homozygote carrier (lower risk or protective haplotype) (Table 1) is calculated as follows: βVKA homozygote = 2βVKA – 2βSEA, where βSEA is less than zero.

#### Multiple testing

The associations of individual amino acids, positions, and haplotypes with all outcome variables were systematically tested in univariate and multivariate analyses. Correction for multiple testing in univariate analyses was carried out using the Benjamini‐Hochberg false discovery rate method.

#### Tests of correlation

**Figure 1:**
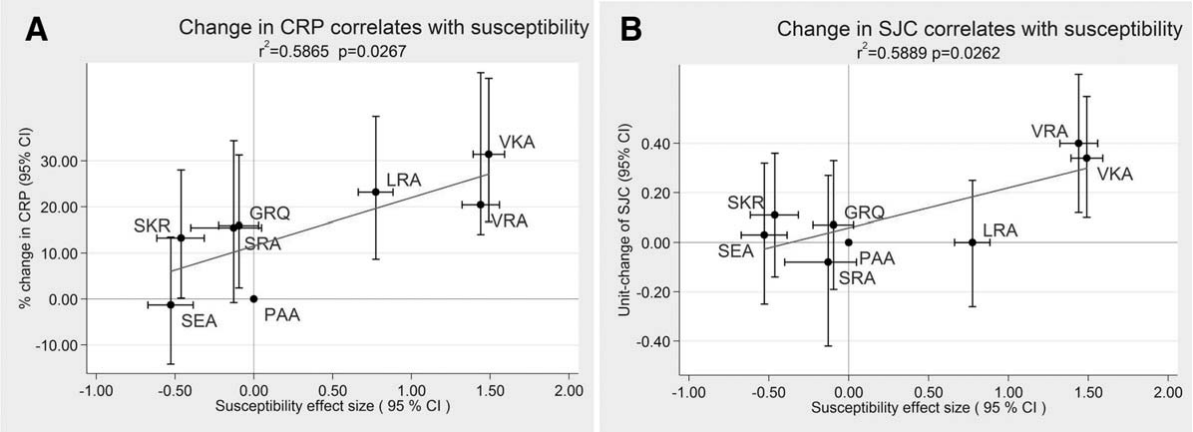
Correlation between genetic markers of susceptibility to rheumatoid arthritis (RA) and nonradiographic measures of outcome. The y‐axes show the effect sizes of HLA–DRB1 haplotypes for their association with nonradiographic measures of RA outcome as determined in this study (change in C‐reactive protein [CRP] level in **A**; change in swollen joint count [SJC] in **B**). The x‐axes show the effect sizes of the same haplotypes for their association with RA susceptibility (natural logarithm of odds ratios as reported in ref. 3). Linear regression was applied to test for an association between these effect sizes. Three‐letter haplotype names are based on combinations of amino acids at HLA–DRB1 positions 11, 71, and 74. 95% CI = 95% confidence interval.

Tests of correlation between measures of radiographic damage (assessed by the Larsen score 18) and nonradiographic measures of disease outcome/activity (the SJC, TJC, DAS28, and HAQ score) were performed with Spearman's rho (non‐normally distributed variables). Correlation between effect sizes of regression analyses (genetic markers of RA susceptibility versus nonradiographic measures of disease outcome) was assessed with linear regression (Figure 1).

#### Mediation analysis

**Figure 2:**
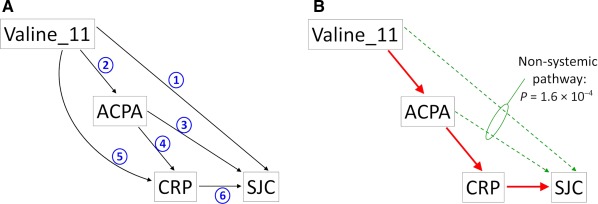
Directed acyclic graphs and results from the mediation analysis. **A,** Hypothetical pathways that lead genetic factors encoding, for example, valine at position 11 of HLA–DRB1, to modulate disease activity/outcome (in this case, the swollen joint count [SJC]). **Numbered arrows** indicate plausible directions of the effect, based on observations of biologic phenomena and chronology. Genetic factors are present at birth, before the appearance of anti–citrullinated protein antibodies (ACPAs), which precede disease onset and inflammation. **B,** Results from the mediation analysis (see text). ACPAs and C‐reactive protein (CRP) level are the main mediators of the effect of valine at position 11 on SJC (systemic inflammatory pathway in red). However, part of the effect of valine at position 11 is mediated independently of CRP level (green nonsystemic pathway).

Mediation analysis was performed to evaluate the extent to which genetic effects on disease outcome could be explained by their effects on intermediate parameters (ACPA status, CRP level). ACPA status (seropositive/seronegative) was determined with the second‐generation CCP2 assay and therefore corresponds to anti–cyclic citrullinated peptide antibody status. First, we drew directed acyclic graphs representing our working hypothesis (Figure 2). Second, a GLLAMM was used to fit models that could be considered causal (i.e., all paths from predictors to outcome consisted only of forward arrows) as described above (orthogonal polynomials of age and disease duration as covariates), either with a unique predictor variable (univariate) or several predictor variables (multivariate), to determine their relative roles (independent effects or not). The β coefficients along with their 95% confidence intervals (95% CIs) and *P* values were used to assess the significance and importance of each predictor variable and pathway (arrows in the acyclic graphs).

## RESULTS

A total of 2,158 inflammatory polyarthritis patients for whom we had data on genotype and the variables of interest were included from the NOAR cohort, and 329 RA patients were included from the ERAS cohort. Demographic and clinical characteristics of the patients are listed in Table 2. Of the patients studied in the NOAR cohort, 77% satisfied the ACR 1987 revised criteria for RA 13, compared with 96% of patients in the ERAS cohort. There was an important difference in the proportion of ACPA‐positive patients between the NOAR (33%) and ERAS (90%) cohorts; this was mainly because NOAR is a primary care inception cohort recruiting all patients with inflammatory polyarthritis within a defined geographic area 19, 20, including those with very low disease severity. As a tertiary care cohort, ERAS preferentially recruits patients with higher disease severity; the DAS28, CRP level, and HAQ score were higher in the ERAS cohort than in the NOAR cohort (Table 2).

**Table 2:** Characteristics of the patients in the NOAR and ERAS cohorts^a^

Characteristic	NOAR cohort (n = 2,158)	ERAS cohort (n = 329)
Recruitment years	1990–2008	1986–1999
Year of final follow‐up	2011	2005
Satisfied ACR 1987 revised criteria for RA during follow‐up, no. (%)	1,951 (77)	316 (96)
Women, no. (%)	1,657 (65)	220 (67)
Age at symptom onset	55 (43–67)	54 (44–62)
Duration of symptoms at baseline, months	6 (3–12)	6 (3–11)
Duration of follow‐up, years	3 (1–5)	7 (3–11)
Observations per patient		
TJC and SJC	3 (1–5)	7 (3–11)
CRP	3 (1–5)	–
ESR	–	7 (3–11)
DAS28	3 (1–5)	7 (3–11)
HAQ	2 (1–4)	7 (3–11)
Ever seropositive for ACPAs (NOAR cohort) or RF (ERAS cohort), no. (%)	713 (33)	297 (90)
Patients with at least 1 copy of SE, no. (%)	1,556 (62)	260 (79)
DAS28 at baseline	3.76 (2.79–4.78)	5.06 (4.19–5.84)
DAS28 at 5 years	2.71 (2.02–3.81)	4.21 (2.87–5.13)
CRP at baseline, mg/liter	8 (2–19.3)	–
CRP at 5 years, mg/liter	6.3 (0–14.1)	–
ESR at baseline, mm/hour	–	36 (18–59)
ESR at 5 years, mm/hour	–	22 (10–42)
HAQ score at baseline	0.875 (0.25–1.5)	1 (0.625–1.6875)
HAQ score at 5 years	0.875 (0.125–1.625)	1 (0.25–1.625)

a All were successfully genotyped patients for whom we had data on the Disease Activity Score in 28 joints (DAS28) and the Health Assessment Questionnaire (HAQ) score. Except where indicated otherwise, values are the median (interquartile range). NOAR = Norfolk Arthritis Register; ERAS = Early Rheumatoid Arthritis Study; ACR = American College of Rheumatology; RA = rheumatoid arthritis; TJC = tender joint count; SJC = swollen joint count; CRP = C‐reactive protein; ESR = erythrocyte sedimentation rate; ACPAs = anti–citrullinated protein antibodies; RF = rheumatoid factor; SE = shared epitope.

Using the same cohorts, we have recently shown that HLA–DRB1 amino acid positions associated with RA susceptibility are also associated with radiographic damage as measured by the Larsen score 3. Since a certain level of correlation is expected between radiographic and nonradiographic measures of disease outcome, we first produced scatterplots of the Larsen score and nonradiographic measures of outcome in the NOAR cohort (see Supplementary Figure 1, available on the *Arthritis & Rheumatology* web site at http://onlinelibrary.wiley.com/doi/10.1002/art.39780/abstract). The graphic pattern shows a poor correlation, which was confirmed by a low Spearman's rho (for TJC with Larsen score, ρ = 0.06; for SJC with Larsen score, ρ = 0.30; for HAQ score with Larsen score, ρ = 0.30; for DAS28 with Larsen score, ρ = 0.29). Therefore, we concluded that the Larsen score is not interchangeable with nonradiographic measures of outcome; genetic factors associated with the Larsen score will not automatically be shared with nonradiographic measures.

We then tested the association of HLA–DRB1 amino acid positions conferring susceptibility with nonradiographic measures of outcome in the NOAR cohort. Results are presented for patients with inflammatory polyarthritis in paragraphs and tables cited below. In univariate analysis, effect sizes are compared to all other amino acids at that position. In multivariate analysis, effect sizes are compared to the reference group (haplotype PAA).

### CRP levels

Among all amino acids tested in univariate analysis, valine at position 11 showed the strongest association, with an increase of 16.27% in the CRP level per copy (*P* = 2.21 × 10^−6^) (Table 3). Amino acids at position 11 were all incorporated in the same multivariate model; position 11 overall was associated with CRP level (*P* = 1.26 × 10^−6^) (see Supplementary Table 1, available on the *Arthritis & Rheumatology* web site at http://onlinelibrary.wiley.com/doi/10.1002/art.39780/abstract). Additionally, univariate analysis showed that several amino acids at position 71 were significantly associated with CRP levels.

**Table 3:** Univariate analysis of amino acids and inflammatory polyarthritis nonradiographic disease outcome measures in the Norfolk Arthritis Register cohort*

Amino acid and position	TJC		SJC		CRP level		DAS28		HAQ score	
	β coefficient (95% CI)†	*P*	β coefficient (95% CI)†	*P*	Percent change (95% CI)	*P*	β coefficient (95% CI)†	*P*	β coefficient (95% CI)†	*P*
Valine 11	−0.03 (−0.20, 0.15)	0.778	0.30 (0.17, 0.43)	7.51 × 10^−6^‡	16.27 (9.23, 23.8)	2.21 × 10^−6^‡	0.10 (0.04, 0.16)	0.002‡	0.02 (0.00, 0.04)	0.044
Leucine 11	−0.16 (−0.38, 0.06)	0.157	−0.18 (−0.34, −0.01)	0.042	6.08 (−2.64, 15.58)	0.177	−0.02 (−0.11, 0.06)	0.607	0.02 (−0.01, 0.04)	0.212
Aspartic acid 11	0.07 (−0.52, 0.66)	0.817	0.31 (−0.12, 0.74)	0.163	2.66 (−17.55, 27.81)	0.815	−0.00 (−0.23, 0.23)	0.987	−0.08 (−0.19, 0.03)	0.161
Proline 11	0.13 (−0.11, 0.37)	0.293	−0.10 (−0.29, 0.08)	0.276	−13.36 (−20.93, −5.07)	0.002‡	−0.04 (−0.13, 0.06)	0.448	−0.03 (−0.07, 0.00)	0.044
Glycine 11	−0.07 (−0.33, 0.18)	0.575	−0.08 (−0.26, 0.10)	0.409	0.66 (−7.90, 10.02)	0.884	−0.02 (−0.11, 0.08)	0.746	0.02 (−0.01, 0.05)	0.248
Serine 11	0.07 (−0.09, 0.23)	0.376	−0.11 (−0.23, 0.01)	0.075	−10.17 (−15.31, −4.72)	4 × 10^−4^‡	−0.06 (−0.12, 0.00)	0.052	−0.02 (−0.04, 0.00)	0.033
Lysine 71	0.02 (−0.17, 0.22)	0.829	0.18 (0.04, 0.31)	0.011	7.07 (0.26, 14.35)	0.042	0.05 (−0.02, 0.12)	0.138	0.01 (−0.01, 0.03)	0.218
Arginine 71	−0.10 (−0.28, 0.07)	0.252	−0.05 (−0.17, 0.07)	0.431	7.43 (1.23, 14.01)	0.018‡	0.00 (−0.06, 0.06)	0.944	0.02 (0.00, 0.04)	0.018
Alanine 71	0.15 (−0.11, 0.41)	0.247	−0.15 (−0.35, 0.05)	0.147	−15.90 (−23.64, −7.38)	4 × 10^−4^‡	−0.05 (−0.15, 0.05)	0.297	−0.06 (−0.09, −0.02)	0.002‡
Glutamic acid 71	0.04 (−0.24, 0.32)	0.783	−0.10 (−0.30, 0.11)	0.355	−16.73 (−24.87, −7.71)	5 × 10^−4^‡	−0.07 (−0.18, 0.04)	0.194	−0.06 (−0.09, −0.03)	4 × 10^−4^‡
Alanine 74	0.01 (−0.16, 0.17)	0.943	0.06 (−0.07, 0.18)	0.401	0.98 (−4.94, 7.28)	0.751	0.04 (−0.03, 0.10)	0.260	−0.01 (−0.03, 0.01)	0.308
Glutamic acid 74	0.12 (−0.22, 0.46)	0.490	0.08 (−0.18, 0.33)	0.559	−1.14 (−12.29, 11.44)	0.851	−0.05 (−0.18, 0.08)	0.434	−0.01 (−0.06, 0.04)	0.754
Arginine 74	0.03 (−0.21, 0.27)	0.827	−0.03 (−0.20, 0.15)	0.763	−3.20 (−11.08, 5.38)	0.453	0.00 (−0.09, 0.09)	0.913	0.00 (−0.02, 0.03)	0.898
Glutamine 74	−0.07 (−0.33, 0.19)	0.590	−0.06 (−0.24, 0.12)	0.497	0.92 (−7.70, 10.34)	0.841	−0.01 (−0.11, 0.09)	0.850	0.02 (−0.01, 0.05)	0.202
Leucine 74	−0.16 (−0.66, 0.33)	0.515	−0.26 (−0.65, 0.13)	0.188	6.35 (−13.03, 30.06)	0.548	−0.16 (−0.37, 0.04)	0.115	0.01 (−0.05, 0.08)	0.676

* An additive model of association was assumed. Statistical effects are reported for 1 copy of every allele. In univariate analysis of amino acids, each individual amino acid is compared to all other amino acids in that position. Multivariate analysis of individual amino acid positions 11, 71, and 74 can be found in Supplementary Table 1 (available on the *Arthritis & Rheumatology* web site at http://onlinelibrary.wiley.com/doi/10.1002/art.39780/abstract). TJC = tender joint count; 95% CI = 95% confidence interval; SJC = swollen joint count; CRP = C‐reactive protein; DAS28 = Disease Activity Score in 28 joints; HAQ = Health Assessment Questionnaire.

† The β coefficient represents an equivalent change in units of the variable of interest.

‡ Significant following Benjamini‐Hochberg correction for multiple testing.

Univariate analysis showed that the valine‐containing haplotypes VKA and VRA were associated with 16.00% (*P* = 4 × 10^−4^) and 15.30% (*P* = 0.008) increases, respectively, in CRP level per copy (see Supplementary Table 2, http://onlinelibrary.wiley.com/doi/10.1002/art.39780/abstract). The serine‐containing haplotype SEA was associated with a decrease of 17.81% (*P* = 0.001) in CRP levels. When all haplotypes were incorporated in a multivariate model, VKA and VRA retained their associations. The 16‐haplotype model was significantly associated with CRP levels (*P* = 2.2 × 10^−6^) (Table 4). When effect sizes for association with CRP level were plotted against effect sizes for susceptibility, a significant correlation was observed (r^2^ = 0.587, *P* = 0.027) (Figure 1A), indicating that haplotypes that predict disease susceptibility also predict the level of systemic inflammation.

**Table 4:** Multivariate analysis of 16‐haplotype classification and inflammatory polyarthritis nonradiographic disease outcome measures in the Norfolk Arthritis Register cohort^a^

	TJC		SJC		CRP level		DAS28		HAQ score	
	β coefficient (95% CI)^b^	*P*	β coefficient (95% CI)^b^	*P*	Percent change (95% CI)	*P*	β coefficient (95% CI)^b^	*P*	β coefficient (95% CI)^b^	*P*
Overall model	–	0.5189	–	0.0017	–	2.2 × 10^−6^	–	0.0207	–	0.0002
Haplotype name^c^										
VKA	−0.17 (−0.50, 0.16)	0.325	0.34 (0.10, 0.59)	0.006	31.46 (16.76, 48.01)	6.19 × 10^−6^	0.11 (−0.01, 0.23)	0.064	0.07 (0.03, 0.11)	0.002
VRA	−0.06 (−0.43, 0.32)	0.756	0.40 (0.12, 0.68)	0.005	30.44 (14.00, 49.25)	1.1 × 10^−4^	0.20 (0.06, 0.34)	0.005	0.08 (0.03, 0.12)	0.003
LRA	−0.32 (−0.65, 0.01)	0.058	0.00 (−0.26, 0.25)	0.977	23.21 (8.66, 39.70)	0.001	0.03 (−0.10, 0.15)	0.668	0.05 (0.01, 0.09)	0.012
PAA	Referent	–	Referent	–	Referent	–	Referent	–	Referent	–
GRQ	−0.27 (−0.61, 0.08)	0.126	0.07 (−0.19, 0.33)	0.607	15.97 (2.42, 31.32)	0.019	0.02 (−0.11, 0.15)	0.760	0.08 (0.03, 0.13)	0.001
SRA	0.03 (−0.38, 0.44)	0.895	−0.08 (−0.42, 0.27)	0.672	15.49 (−0.78, 34.43)	0.063	−0.02 (−0.18, 0.14)	0.829	0.05 (−0.01, 0.11)	0.098
SKR	−0.14 (−0.48, 0.20)	0.412	0.11 (−0.14, 0.36)	0.387	13.28 (0.22, 28.05)	0.046	0.05 (−0.08, 0.18)	0.471	0.06 (0.02, 0.11)	0.008
SEA	−0.10 (−0.48, 0.28)	0.617	0.03 (−0.25, 0.32)	0.822	−1.30 (−14.18, 13.50)	0.854	−0.02 (−0.16, 0.13)	0.808	0.00 (−0.04, 0.05)	0.896

a Haplotypes with a study population frequency of <5% are excluded. Therefore, only the 8 most common haplotypes are presented. An additive model of alleles (i.e., haplotypes) was created. Statistical effects are reported for 1 copy of every allele. In multivariate analysis of haplotypes, each haplotype is adjusted in the multivariate model, using the haplotype PAA as the referent. Univariate analysis of the associations between the 16‐haplotype model and inflammatory polyarthritis disease outcome measures is shown in Supplementary Table 2 (available on the *Arthritis & Rheumatology* web site at http://onlinelibrary.wiley.com/doi/10.1002/art.39780/abstract). TJC = tender joint count; 95% CI = 95% confidence interval; SJC = swollen joint count; CRP = C‐reactive protein; DAS28 = Disease Activity Score in 28 joints; HAQ = Health Assessment Questionnaire.

b The β coefficient represents an equivalent change in units of the variable of interest.

c See Table 1 for derivation of haplotype names.

### SJC

Valine at position 11 was associated with an increase of 0.30 swollen joints per copy (*P* = 7.51 × 10^−6^) (Table 3). Position 11 was significantly associated with the SJC (*P* = 0.002). The valine‐containing haplotypes VKA and VRA were associated with increases of 0.29 (*P* = 0.001) and 0.35 (*P* = 0.002) swollen joints per copy, respectively (see Supplementary Table 2, http://onlinelibrary.wiley.com/doi/10.1002/art.39780/abstract). The multivariate model of all haplotypes was significantly associated with the SJC (*P* = 0.0017), and the associations of VKA/VRA retained significance (Table 4). Effect sizes in Results tables are reported per copy of every allele, compared to a reference group. Effect sizes are larger if expressed for homozygote carriers (e.g., the effect sizes for a VKA and VRA homozygote compared to the reference PAA homozygote are 0.68 and 0.80 joints, respectively). The 16‐haplotype model demonstrated significant correlation between susceptibility and SJC (r^2^ = 0.589, *P* = 0.026) (Figure 1B).

### TJC

There were no associations between individual amino acids/haplotypes and the TJC.

### DAS28

Valine at position 11 was associated with an increase of 0.10 DAS28 units per copy (*P* = 0.002) (Table 3). The multivariate model of 16 haplotypes was significantly associated with the DAS28 (*P* = 0.0207), with the valine‐containing haplotype VRA being associated with an increase of 0.20 DAS28 units per copy (*P* = 0.005) (Table 4). Effect sizes are larger if expressed for homozygote carriers and if the reference group is a homozygote protective haplotype; the average difference in the DAS28 between a VRA homozygote carrier and an SEA homozygote carrier is 0.44 DAS28 units.

### HAQ score

Multivariate analysis of all 16 haplotypes showed increases of 0.07 and 0.08 HAQ score units per copy in the haplotypes VKA and VRA, respectively, with the model overall being associated with HAQ scores (*P* = 0.0002) (Table 4).

### Findings of subanalysis in RA patients and adjustment for treatment

We conducted a subanalysis in patients satisfying the ACR 1987 revised criteria for RA 13 (see Supplementary Table 3, http://onlinelibrary.wiley.com/doi/10.1002/art.39780/abstract). Results for CRP level were similar in NOAR patients with RA (Supplementary Table 3). For example, the VKA haplotype was associated with a 33.12% increase in CRP level per copy (*P* = 2.44 × 10^−5^) in multivariate analysis. The associations reported above for the 16‐haplotype model in patients with inflammatory polyarthritis remained significant after adjustment for treatment (data not shown).

### Validation with the ERAS cohort

Results for SJC and the marker of inflammation used to calculate the DAS28, in this case the ESR, were replicated within the ERAS cohort (see Supplementary Table 4, http://onlinelibrary.wiley.com/doi/10.1002/art.39780/abstract). Valine at position 11 was significantly associated with an increase of 0.82 swollen joints per copy (*P* = 4.55 × 10^−6^), and the haplotype VKA was significantly associated with a 10.94% increase in the ESR (*P* = 5 × 10^−5^).

### Findings of mediation analysis

Figure 2A shows a directed acyclic graph summarizing plausible biologic paths from genetic markers within HLA–DRB1 (valine at position 11 [Val^11^]) to disease activity/outcome (measured, for example, by the SJC). First, we tested the association of Val^11^ and CRP level with SJC in a bivariate analysis; this investigates the effect of Val^11^ when not mediated through CRP level (indicated by arrows 1–3 in Figure 2A) and the effect of other factors (i.e., other genetic or environmental factors) that are mediated by CRP level (indicated by arrow 6 in Figure 2A; not including effects indicated by arrow 5). We found that both Val^11^ (β coefficient of 0.39 [95% CI 0.19, 0.59], *P* = 1.6 × 10^−4^) and CRP level (β coefficient of 0.03 [95% CI 0.02, 0.03], *P* = 2.8 × 10^−22^) were independently associated with SJC. Therefore, Val^11^ is also mediating its effect independently of the CRP level through a nonsystemic pathway (green pathway in Figure 2B).

In order to investigate whether the nonsystemic effect of Val^11^ on SJC is direct (arrow 1 in Figure 2A) or indirect through ACPA status (arrows 2 and 3 in Figure 2A), we added ACPA status into the model (trivariate analysis, including Val^11^, CRP level, and ACPA status). We obtained an association for Val^11^ with SJC that was almost significant (β coefficient of 0.25 [95% CI −0.00, 0.50], *P* = 0.051), but there was no significant association of ACPA status with SJC (β coefficient of 0.22 [95% CI −0.15, 0.59], *P* = 0.24), indicating that it is not possible to determine whether the nonsystemic effect of Val^11^ is direct or mediated by ACPA status (green arrows in Figure 2B). However, in view of the 95% CIs and *P* values reported herein, a direct effect of Val^11^ on SJC is likely to explain the significant association of Val^11^ with SJC observed in the bivariate analysis. Next, we investigated the association of Val^11^ with CRP level using a bivariate analysis: 2 independent variables (ACPA status and Val^11^) for 1 outcome variable (ln[CRP level + 1]). This compares 2 pathways, indicated by arrow 5 versus arrows 2 and 4 in Figure 2A. We did not detect any direct effect of Val^11^ on CRP level (β coefficient of 0.04 [95% CI −0.04, 0.11], *P* = 0.32), but there was a significant effect of ACPA status on CRP level (β coefficient of 0.72 [95% CI 0.61, 0.82], *P* = 3.6 × 10^−38^), indicating that most of the effect of Val^11^ on CRP level is mediated by ACPA status (systemic inflammatory pathway).

The effect of removing nonsignificant pathways from Figure 2A is shown in Figure 2B. The effect of Val^11^ on disease activity/outcome is mainly mediated by ACPA status, the effect of which is mainly mediated by systemic inflammation (pathway indicated by red arrows in Figure 2B), but the effect of Val^11^ on disease activity/outcome is also mediated through a nonsystemic pathway (green pathway in Figure 2B), most likely independently of ACPA status.

Finally, we decided to test whether the effect of Val^11^ could be detected independently in ACPA‐positive patients and in ACPA‐negative patients. This stratification analysis is equivalent to an adjustment for ACPA status. As we have shown above in the mediation analysis, ACPA status is a path variable from Val^11^ to SJC. It is well known that adjusting for a known path variable will remove the association, unless Val^11^ also develops its effect on SJC independently of ACPA status. As we have shown in the mediation analysis, this is a plausible hypothesis, but our study lacked sufficient power to provide a clear answer. The results of the analysis after stratification by ACPA status are presented in Supplementary Table 5 (http://onlinelibrary.wiley.com/doi/10.1002/art.39780/abstract). Although the association of genetic factors reached nominal significance in a few instances (for the association of Val^11^ with SJC in ACPA‐negative patients, β coefficient of 0.18 [95% CI 0.01, 0.35], *P* = 0.038), most associations did not remain significant after stratification by ACPA status. This pattern of association (highly significant *P* value before stratification, lack of significance after stratification) confirms that ACPA status is an important path variable in the etiology of clinical inflammation, but this analysis is inconclusive with regard to a minor independent effect of genetic factors on SJC (due to an underpowered study design).

## DISCUSSION

In this study, we performed a detailed examination of associations of HLA–DRB1 positions 11/13, 71, and 74 with inflammatory polyarthritis and RA disease activity and outcome measures (HAQ score and DAS28 and its subcomponents). We demonstrate that amino acids at position 11/13, outside of the classical SE, show the strongest association with clinical and laboratory measures of inflammation. Valine at position 11 shows the greatest effect, while serine at the same position is protective; amino acids at position 71 also contribute. Indeed, analysis showed that haplotypes predicting disease susceptibility also predict levels of systemic inflammation and clinical inflammation (Figures 1A and B), and the same risk hierarchy of effect sizes is observed for susceptibility and for measures of inflammation.

The effect of valine at position 11 on SJC (clinical inflammation) is mainly mediated by ACPA status and systemic (or laboratory‐determined) inflammation (CRP level). However, we could also show that this association is independent of CRP level; this is an important finding, since several studies have now shown that RA can be active with no elevation of CRP level and can progress in the absence of detectable systemic inflammation 7, 8. In the absence of clinical signs of inflammation (no swollen joints), radiographic progression was shown to be similar between patients with normal CRP levels and those with CRP levels indicating active disease; however, in the absence of laboratory‐determined active inflammation (CRP level <1 mg/dl), SJC determined radiographic progression 8. Therefore, it seems that clinical inflammation rather than laboratory‐determined inflammation is associated with radiographic progression and mortality 8.

We have shown in a previous study 3 that RA genetic susceptibility markers within HLA–DRB1 predict radiographic damage in RA. In the present study, we tested hypotheses regarding the mechanisms of action of genetic variations and showed that the same genetic markers within HLA–DRB1 have an effect on clinical inflammation (SJC) and laboratory‐determined or systemic inflammation (CRP level) independently of each other. Therefore, amino acid positions 11/13, 71, and 74 within HLA–DRB1 are likely to mediate their effect on radiographic outcome through at least 2 distinct inflammatory pathways. Drawing the directed acyclic graph differently by substituting SJC for CRP level, assuming that clinical inflammation (SJC) causes laboratory‐determined inflammation (CRP level), did not affect our conclusion (data not shown).

Only 33% of patients are positive for ACPAs in the NOAR cohort, while the proportion is higher in the ERAS cohort (90%). This is mainly because NOAR is a primary care inception cohort recruiting all patients who have had ≥2 swollen joints for >4 weeks within a defined geographic area. It is worth noting that the level of recruitment is very high. Consequently, inflammatory polyarthritis and RA patients with very low disease severity (and more likely to be ACPA negative) are also included in the NOAR cohort. Subsequently, 32% of inflammatory polyarthritis patients have been found to have drug‐free remission of their disease by the 3‐year follow‐up visit 19, which indicates a high proportion of patients with disease of low severity. The tertiary care ERAS cohort preferentially recruits patients with higher disease severity; the DAS28, CRP level, and HAQ score are higher in the ERAS cohort than in the NOAR cohort (Table 2). It is also noteworthy that the French Étude et Suivi des Polyarthrites Indifférenciées Récentes (ESPOIR) cohort, an inception cohort similar to the NOAR cohort but consisting of RA patients recruited from tertiary care, has a similar proportion of ACPA‐positive patients (38.7%) 21. Having a large proportion of ACPA‐negative patients in the NOAR cohort represents a strength of our study, as it provides sufficient power to determine the role of ACPAs in the path to clinical and laboratory‐determined inflammation.

It is remarkable that CRP levels are controlled by RA susceptibility loci within the HLA region in active disease. CRP levels are known to be partially genetically determined (but not by HLA genes) 22, 23, and haplotypes of common single‐nucleotide polymorphisms (SNPs) within the CRP locus have been reported in some 24, but not all, studies to correlate with CRP levels in RA patients. For example, we were unable to replicate the correlation of CRP haplotypes with CRP levels in a cohort of RA patients with high disease activity (the Biologics in Rheumatoid Arthritis Genetics and Genomics Study Syndicate cohort) 25, possibly because the study was underpowered (fewer than 600 patients). The use of CRP levels to guide treatment decisions has been questioned 24, since carriers of low CRP–associated genetic variants could still have active disease and show disease progression in the absence of laboratory‐determined inflammation 7, 8. As a possible genetic explanation of this observation, we show in the present study that RA susceptibility loci within the HLA region are correlated with the SJC (clinical inflammation) independently of the CRP level (laboratory‐determined inflammation). A limitation of the current study is that haplotypes of common SNPs within the CRP locus were not available in samples from the NOAR and ERAS cohorts, so we could not undertake a formal comparison of the effect sizes of CRP haplotypes and HLA haplotypes on CRP levels within the same cohort.

The findings of the current study support and build on previous findings that HLA–DRB1 susceptibility risk haplotypes encompassing the same 3 positions also correlate with radiographic and mortality data 3. Neither markers of inflammation nor the DAS28 and its components were analyzed in that study. The current findings may suggest a mechanistic explanation, because we have shown that CRP levels are particularly influenced by the 16‐haplotype model. There is a well‐established relationship between time‐integrated CRP levels and radiographic progression in RA patients 6; our model suggests that the effect of genetic determinants of radiographic damage is mediated by inflammation. The association between the 16‐haplotype model and the SJC likely confirms the SJC as a surrogate indicator of active synovitis/inflammation.

Stronger associations were found between the 16‐haplotype model and objective variables studied, such as CRP levels and SJC. The results with the TJC (a more subjective variable) and with the DAS28 (a composite variable) reflect that the inclusion of subjective variables such as TJC diminishes the association between the DAS28 and disease severity; this finding is corroborated by an independent study 5. In the future, TJC may even be removed altogether from outcome calculations, as it may not be a sensitive enough indicator of joint damage or disease activity.

A greater understanding of genetic susceptibility to RA and its adverse disease outcomes may lead to a stratified medicine approach 26 with 16 risk categories, as patients with increased susceptibility to developing RA are also more likely to have a worse disease course, requiring earlier and more aggressive intervention. The current study shows that genetic changes influence levels of inflammation, which in turn lead to joint damage. The “treat‐to‐target” strategies 27 have shown that control of disease activity, as measured by composite scoring methods, has resulted in improving outcomes, but the current study suggests that controlling objective measures of inflammation (CRP levels and SJC) may be the key factor.

In conclusion, our findings demonstrate an association of amino acids at position 11/13 within the HLA–DRB1 gene (but outside the SE), and also of haplotypes involving amino acid positions 11/13, 71, and 74, with objective disease outcome measures. In addition, our findings describe a genetic basis for the increased inflammatory response that is known to lead to radiographic progression in patients with inflammatory polyarthritis or RA.

## AUTHOR CONTRIBUTIONS

All authors were involved in drafting the article or revising it critically for important intellectual content, and all authors approved the final version to be published. Dr. Barton had full access to all of the data in the study and takes responsibility for the integrity of the data and the accuracy of the data analysis.

### Study conception and design

Viatte, Symmons, Young, Macgregor, Barton.

### Acquisition of data

Van Sijl, Silva‐Fernandez, Symmons, Young, Macgregor, Barton.

### Analysis and interpretation of data

Ling, Viatte, Lunt, Symmons, Young, Macgregor, Barton.

## Supporting information

SUPPLEMENTARY FIGURE 1 Scatter plot of non‐radiographic measures of IP outcome show a poor correlation with a radiographic measure (Larsen score). TJC, SJC, HAQ score and DAS28 of NOAR patients at year 5 were compared with their Larsen score (one dot represents at least one patient).
Supplementary Table 1. Multivariate analysis of AAs and non‐radiographic disease outcome measures in NOAR patients with IP.
Supplementary Table 2. Univariate analysis of 16‐haplotype classification and non‐radiographic disease outcome measures in NOAR patients with IP.
Supplementary Table 3. Sub‐analysis in NOAR patients satisfying ACR 1987 criteria.
Supplementary Table 4. Validation of IP disease outcome measures in ERAS cohort.
Supplementary Table 5. Sub‐analysis in NOAR patients with IP by ACPA status (seropositive and seronegative).
