# Supplementary material for: HLA–DRB1 Amino Acid Positions 11/13, 71, and 74 Are Associated With Inflammation Level, Disease Activity, and the Health Assessment Questionnaire Score in Patients With Inflammatory Polyarthritis
Source: Arthritis Rheumatol. 2016 Oct 27;68(11):2618–28. doi: 10.1002/art.39780 (PMC5244675; doi:10.1002/art.39780)
Supplement: Supplementary file 1 — SUPPLEMENTARY FIGURE 1 Scatter plot of non‐radiographic measures of IP outcome show a poor correlation with a radiographic measure (Larsen score). TJC, SJC, HAQ score and DAS28 of NOAR patients at year 5 were compared with their Larsen score (one dot represents at least one patient). Supplementary Table 1. Multivariate analysis of AAs and non‐radiographic disease outcome measures in NOAR patients with IP. Supplementary Table 2. Univariate analysis of 16‐haplotype classification and non‐radiographic disease outcome measures in NOAR patients with IP. Supplementary Table 3. Sub‐analysis in NOAR patients satisfying ACR 1987 criteria. Supplementary Table 4. Validation of IP disease outcome measures in ERAS cohort. Supplementary Table 5. Sub‐analysis in NOAR patients with IP by ACPA status (seropositive and seronegative). [file ART-68-2618-s001.docx]

**SUPPLEMENTARY FIGURE 1**


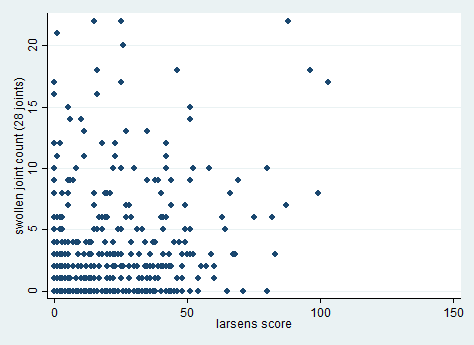


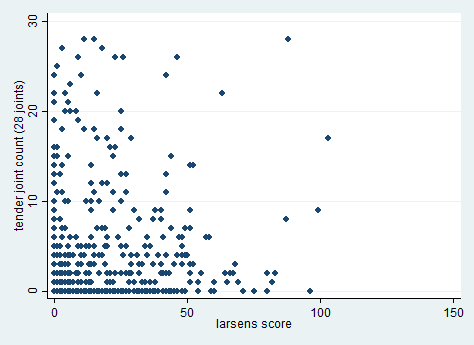


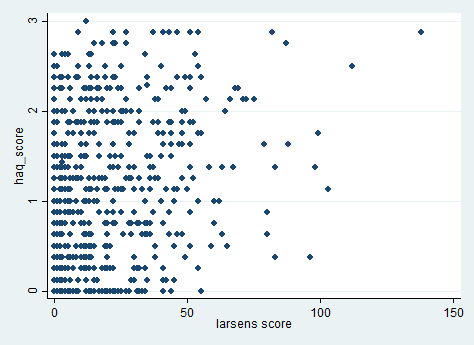


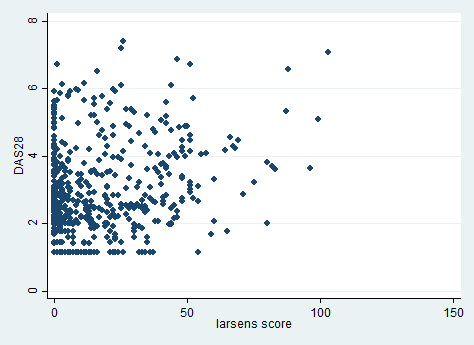


**Scatter plot of non-radiographic measures of IP outcome show a poor correlation with a radiographic measure (Larsen score).**

TJC, SJC, HAQ score and DAS28 of NOAR patients at year 5 were compared with their Larsen score (one dot represents at least one patient).

**SUPPLEMENTARY TABLES**

**Supplementary Table 1. Multivariate analysis of AAs and non-radiographic disease outcome measures in NOAR patients with IP.**

| *Amino acid* | **TJC** | | **SJC** | | **CRP** | | **DAS28** | | **HAQ** | |
| --- | --- | --- | --- | --- | --- | --- | --- | --- | --- | --- |
|  | *Coeff (95% CI)* | *p-value* | *Coeff (95% CI)* | *p-value* | *% change (95% CI)* | *p-value* | *Coeff (95% CI)* | *p-value* | *Coeff (95% CI)* | *p-value* |
| **Position 11**  Valine  Leucine  Asp. acid  Proline  Glycine  Serine | 0.05 (-0.24, 0.34)  -0.07 (-0.39, 0.24)  0.14 (-0.50, 0.78)  0.19 (-0.15, 0.52)  (Reference group)  0.12 (-0.16, 0.39) | **0.5999**  0.724  0.660  0.659  0.271  0.413 | 0.28 (0.08, 0.49)  -0.08 (-0.32, 0.15)  0.38 (-0.08, 0.84)  -0.02 (-0.27, 0.23)  (Reference group)  -0.00 (-0.20, 0.20) | **0.0002**  0.006  0.482  0.108  0.881  0.994 | 11.34 (0.83, 22.94)  5.53 (-5.99, 18.47)  3.38 (-18.21, 30.67)  -12.43 (-22.29, -1.31)  (Reference group)  -7.08 (-15.55, 2.24) | **1.26E-06**  0.034  0.362  0.781  0.030  0.132 | 0.09 (-0.02, 0.19)  0.00 (-0.12, 0.12)  0.01 (-0.23, 0.26)  -0.02 (-0.14, 0.11)  (Reference group)  -0.02 (-0.13, 0.08) | **0.0621**  0.103  0.991  0.929  0.807  0.645 | 0.00 (-0.03, 0.04)  0.00 (-0.04, 0.04)  -0.09 (-0.20, 0.03)  -0.04 (-0.08, 0.01)  (Reference group)  -0.02 (-0.05, 0.01) | **0.0345**  0.856  0.946  0.144  0.104  0.183 |
| **Position 71**  Lysine  Arginine  Alanine  Glut. acid | -0.11 (-0.40, 0.18)  -0.18 (-0.45, 0.09)  (Reference group)  -0.10 (-0.46, 0.26) | **0.5921**  0.448  0.186  0.588 | 0.26 (0.04, 0.48)  0.11 (-0.10, 0.32)  (Reference group)  0.05 (-0.22, 0.32) | **0.0520**  0.020  0.296  0.730 | 22.48 (10.08, 36.27)  20.86 (9.36, 33.57)  (Reference group)  -1.10 (-13.48, 13.05) | **4.1E-06**  2E-04*  3E-04*  0.871 | 0.09 (-0.02, 0.20)  0.05 (-0.05, 0.15)  (Reference group)  -0.02 (-0.15, 0.12) | **0.2469**  0.128  0.340  0.816 | 0.06 (0.02, 0.10)  0.06 (0.02, 0.10)  (Reference group)  0.00 (-0.04, 0.05) | **2.86E-05**  0.003  0.001  0.930 |
| **Position 74**  Alanine  Glut. acid  Arginine  Glutamine  Leucine | -0.11 (-0.45, 0.24)  (Reference group)  -0.08 (-0.48, 0.32)  -0.17 (-0.59, 0.24)  -0.27 (-0.85, 0.31) | **0.8834**  0.544  0.702  0.404  0.365 | -0.05 (-0.30, 0.21)  (Reference group)  -0.08 (-0.37, 0.21)  -0.12 (-0.42, 0.18)  -0.32 (-0.77, 0.14) | **0.6152**  0.724  0.577  0.421  0.171 | 1.45 (-10.13, 14.51)  (Reference group)  -1.77 (-14.61, 12.99)  1.99 (-11.64, 17.72)  7.66 (-14.50, 35.56) | **0.9134**  0.816  0.802  0.788  0.530 | 0.06 (-0.07, 0.19)  (Reference group)  0.05 (-0.10, 0.19)  0.04 (-0.11, 0.19)  -0.11 (-0.35, 0.12) | **0.4850**  0.357  0.533  0.621  0.357 | 0.00 (-0.05, 0.05)  (Reference group)  0.01 (-0.04, 0.06)  0.02 (-0.03, 0.08)  0.02 (-0.06, 0.10) | **0.7026**  0.927  0.721  0.389  0.643 |

*Abbreviations*: AA = amino acid. NOAR = Norfolk Arthritis Register. IP = inflammatory polyarthritis. TJC = tender joint count. SJC = swollen joint count. CRP = C-reaction protein. DAS28 = disease activity score of 28 joints. HAQ = Health Assessment Questionnaire. Coeff = β-coefficient – this represents an equivalent change in units of the variable of interest. 95% CI = 95% confidence intervals of β-coefficient value. Asp. acid = aspartic acid. Glut. acid = glutamic acid.

**Supplementary Table 2. Univariate analysis of 16-haplotype classification and non-radiographic disease outcome measures in NOAR patients with IP.**

| *Haplotype* | **TJC** | | **SJC** | | **CRP** | | **DAS28** | | **HAQ** | |
| --- | --- | --- | --- | --- | --- | --- | --- | --- | --- | --- |
|  | *Coeff (95% CI)* | *p-value* | *Coeff (95% CI)* | *p-value* | *% change (95% CI)* | *p-value* | *Coeff (95% CI)* | *p-value* | *Coeff (95% CI)* | *p-value* |
| VKA | -0.01 (-0.25, 0.23) | 0.944 | 0.29 (0.12, 0.46) | 0.001* | 16.00 (6.79, 26.00) | 4E-04* | 0.09 (0.01, 0.18) | 0.027 | 0.03 (-0.00, 0.06) | 0.096 |
| VRA | 0.12 (-0.18, 0.42) | 0.434 | 0.35 (0.13, 0.57) | 0.002* | 15.30 (3.84, 28.01) | 0.008* | 0.19 (0.08, 0.29) | 0.001* | 0.04 (-0.00, 0.07) | 0.055 |
| LRA | -0.17 (-0.40, 0.07) | 0.160 | -0.14 (-0.32, 0.04) | 0.127 | 5.66 (-3.49, 15.67) | 0.233 | -0.02 (-0.11, 0.06) | 0.595 | 0.02 (-0.01, 0.04) | 0.287 |
| PAA | 0.15 (-0.11, 0.40) | 0.265 | -0.13 (-0.33, 0.07) | 0.189 | -16.00 (-23.75, -7.45) | 4E-04* | -0.05 (-0.15, 0.05) | 0.313 | -0.06 (-0.10, -0.03) | 0.001* |
| GRQ | -0.13 (-0.39, 0.13) | 0.336 | -0.06 (-0.25, 0.12) | 0.490 | -0.06 (-8.75, 9.46) | 0.990 | -0.03 (-0.13, 0.07) | 0.595 | 0.03 (0.00, 0.06) | 0.047 |
| SRA | 0.19 (-0.17, 0.54) | 0.303 | -0.26 (-0.57, 0.05) | 0.096 | -1.66 (-13.59, 11.93) | 0.800 | -0.07 (-0.21, 0.07) | 0.307 | -0.01 (-0.05, 0.03) | 0.625 |
| SKR | 0.03 (-0.21, 0.28) | 0.804 | -0.03 (-0.21, 0.15) | 0.743 | -4.29 (-12.22, 4.35) | 0.320 | -0.01 (-0.10, 0.08) | 0.765 | 0.00 (-0.02, 0.03) | 0.829 |
| SEA | 0.08 (-0.22, 0.38) | 0.597 | -0.11 (-0.33, 0.12) | 0.351 | -17.81 (-25.67, -7.28) | 0.001* | -0.07 (-0.18, 0.04) | 0.228 | -0.06 (-0.09, -0.03) | 4E-04* |

**Remains significant following Benjamini-Hochberg correction for multiple testing*

*Abbreviations*: NOAR = Norfolk Arthritis Register. IP = inflammatory polyarthritis. TJC = tender joint count. SJC = swollen joint count. CRP = C-reaction protein. DAS28 = disease activity score of 28 joints. HAQ = Health Assessment Questionnaire. Coeff = β-coefficient – this represents an equivalent change in units of the variable of interest. 95% CI = 95% confidence intervals of β-coefficient value.

Haplotypes with a study population frequency of <5% were excluded from this table. Therefore, only the 8 commonest haplotypes are presented.

**Supplementary Table 3. Sub-analysis in NOAR patients satisfying ACR 1987 criteria.**

1. **Univariate analysis of AAs for non-radiographic disease outcome measures in patients with RA.**

| *Amino acid* | **TJC** | | **SJC** | | **CRP** | | **DAS28** | | **HAQ** | |
| --- | --- | --- | --- | --- | --- | --- | --- | --- | --- | --- |
|  | *Coeff (95% CI)* | *p-value* | *Coeff (95% CI)* | *p-value* | *% change (95% CI)* | *p-value* | *Coeff (95% CI)* | *p-value* | *Coeff (95% CI)* | *p-value* |
| Valine 11 | -0.11 (-0.32, 0.11) | 0.339 | 0.15 (-0.00, 0.30) | 0.055 | 14.66 (6.82, 23.09) | 2E-04 | 0.04 (-0.03, 0.12) | 0.245 | -0.00 (-0.03, 0.02) | 0.842 |
| Leucine 11 | -0.27 (-0.55, 0.01) | 0.059 | -0.22 (-0.43, -0.01) | 0.037 | 4.45 (-42.69, 15.35) | 0.389 | -0.06 (-0.16, 0.04) | 0.212 | 0.02 (-0.01, 0.06) | 0.176 |
| Asp. acid 11 | -0.01 (-0.79, 0.78) | 0.987 | 0.29 (-0.22, 0.80) | 0.268 | 6.68 (-16.92, 37.00) | 0.612 | 0.05 (-0.21, 0.32) | 0.686 | 0.08 (-0.02, 0.17) | 0.109 |
| Proline 11 | 0.16 (-0.16, 0.47) | 0.330 | -0.15 (-0.39, 0.09) | 0.213 | -15.13 (-23.71, -5.59) | 0.003 | -0.03 (-0.14, 0.08) | 0.631 | 0.02 (-0.02, 0.07) | 0.278 |
| Glycine 11 | 0.01 (-0.33, 0.36) | 0.932 | -0.08 (-0.31, 0.15) | 0.483 | 3.49 (-6.68, 14.76) | 0.516 | 0.03 (-0.09, 0.14) | 0.642 | 0.05 (0.01, 0.08) | 0.008 |
| Serine 11 | 0.17 (-0.04, 0.37) | 0.104 | 0.05 (-0.10, 0.20) | 0.538 | -9.66 (-15.66, -3.24) | 0.004 | -0.01 (-0.08, 0.06) | 0.756 | -0.04 (-0.07, -0.02) | 0.001 |
|  | | | | | | | | | | |
| Lysine 71 | 0.07 (-0.18, 0.31) | 0.588 | 0.19 (0.03, 0.36) | 0.020 | 7.29 (-0.45, 15.64) | 0.066 | 0.03 (-0.05, 0.11) | 0.435 | -0.01 (-0.04, 0.02) | 0.532 |
| Arginine 71 | -0.19 (-0.40, 0.03) | 0.088 | -0.07 (-0.22, 0.08) | 0.376 | 8.13 (1.03, 15.73) | 0.024 | 0.00 (-0.07, 0.08) | 0.924 | 0.03 (0.01, 0.06) | 0.010 |
| Alanine 71 | 0.17 (-0.17, 0.50) | 0.333 | -0.18 (-0.43, 0.08) | 0.179 | -17.14 (-25.96, -7.27) | 0.001 | -0.04 (-0.16, 0.07) | 0.465 | 0.01 (-0.04, 0.06) | 0.601 |
| Glut. acid 71 | 0.19 (-0.18, 0.55) | 0.319 | -0.09 (-0.35, 0.16) | 0.475 | -19.29 (-28.49, -8.91) | 0.001 | -0.04 (-0.17, 0.09) | 0.529 | -0.12 (-0.16, -0.07) | 1.72E-07 |
|  | | | | | | | | | | |
| Alanine 74 | -0.11 (-0.32, 0.11) | 0.321 | -0.04 (-0.19, 0.11) | 0.578 | 0.17 (-6.53, 7.34) | 0.962 | -0.02 (-0.09, 0.06) | 0.656 | -0.03 (-0.05, 0.00) | 0.058 |
| Glut. acid 74 | -0.03 (-0.46, 0.39) | 0.884 | -0.13 (-0.42, 0.17) | 0.404 | 2.22 (-10.73, 17.04) | 0.751 | -0.05 (-0.19, 0.10) | 0.534 | 0.02 (-0.03, 0.07) | 0.449 |
| Arginine 74 | 0.20 (-0.11, 0.51) | 0.214 | 0.22 (-0.00, 0.43) | 0.051 | -2.90 (-12.06, 7.20) | 0.560 | 0.04 (-0.06, 0.15) | 0.441 | -0.01 (-0.05, 0.02) | 0.492 |
| Glutamine 74 | 0.00 (-0.34, 0.34) | 0.987 | -0.08 (-0.31, 0.15) | 0.504 | 3.48 (-6.70, 14.77) | 0.517 | 0.03 (-0.09, 0.14) | 0.640 | 0.05 (0.01, 0.08) | 0.006 |
| Leucine 74 | 0.21 (-0.44, 0.87) | 0.519 | 0.08 (-0.44, 0.59) | 0.775 | -10.24 (-30.36, 15.69) | 0.404 | -0.04 (-0.29, 0.21) | 0.777 | -0.01 (-0.08, 0.06) | 0.880 |

1. **Multivariate analysis of AAs and non-radiographic disease outcome measures in patients with RA.**

| *Amino acid* | **TJC** | | **SJC** | | **CRP** | | **DAS28** | | **HAQ** | |
| --- | --- | --- | --- | --- | --- | --- | --- | --- | --- | --- |
|  | *Coeff (95% CI)* | *p-value* | *Coeff (95% CI)* | *p-value* | *% change (95% CI)* | *p-value* | *Coeff (95% CI)* | *p-value* | *Coeff (95% CI)* | *p-value* |
| **Position 11**  Valine  Leucine  Asp. acid  Proline  Glycine  Serine | -0.09 (-0.46, 0.29)  -0.24 (-0.65, 0.17)  0.03 (-0.84, 0.90)  0.13 (-0.30, 0.57)  (Reference group)  0.09 (-0.27, 0.46) | **0.2827**  0.653  0.245  0.947  0.543  0.610 | 0.18 (-0.07, 0.42)  -0.12 (-0.41, 0.16)  0.38 (-0.17, 0.93)  -0.06 (-0.38, 0.25)  (Reference group)  0.10 (-0.14, 0.35) | **0.0786**  0.164  0.401  0.177  0.688  0.418 | 7.88 (-3.83, 21.02)  2.56 (-10.36, 17.35)  4.81 (-19.99, 37.29)  -15.76 (-26.71, -3.19)  (Reference group)  -8.83 (-18.42, 1.89) | **0.0001**  0.196  0.713  0.733  0.016  0.103 | 0.00 (-0.12, 0.13)  -0.08 (-0.22, 0.07)  0.04 (-0.24, 0.32)  -0.05 (-0.20, 0.11)  (Reference group)  -0.03 (-0.16, 0.09) | **0.7228**  0.959  0.289  0.798  0.557  0.597 | -0.04 (-0.08, 0.00)  -0.02 (-0.06, 0.03)  0.05 (-0.05, 0.14)  -0.01 (-0.06, 0.04)  (Reference group)  -0.06 (-0.10, -0.03) | **0.0017**  0.052  0.508  0.338  0.603  0.001 |
| **Position 71**  Lysine  Arginine  Alanine  Glut. acid | -0.08 (-0.46, 0.30)  -0.23 (-0.57, 0.12)  (Reference group)  0.02 (-0.46, 0.50) | **0.3535**  0.675  0.202  0.921 | 0.30 (0.02, 0.58)  0.13 (-0.14, 0.39)  (Reference group)  0.09 (-0.26, 0.44) | **0.0963**  0.037  0.346  0.619 | 23.84 (9.47, 40.09)  22.48 (9.02, 37.59)  (Reference group)  -2.61 (-16.86, 14.09) | **1.54E-05**  0.001  0.001  0.743 | 0.06 (-0.07, 0.19)  0.04 (-0.08, 0.16)  (Reference group)  0.00 (-0.16, 0.17) | **0.7340**  0.351  0.506  0.975 | -0.01 (-0.06, 0.05)  0.01 (-0.04, 0.07)  (Reference group)  -0.11 (-0.17, -0.05) | **1.93E-06**  0.813  0.575  4E-04 |
| **Position 74**  Alanine  Glut. acid  Arginine  Glutamine  Leucine | -0.01 (-0.44, 0.42)  (Reference group)  0.19 (-0.31, 0.69)  0.01 (-0.52, 0.54)  0.20 (-0.55, 0.96) | **0.7434**  0.959  0.463  0.962  0.599 | 0.10 (-0.20, 0.40)  (Reference group)  0.30 (-0.05, 0.65)  0.05 (-0.31, 0.41)  0.15 (-0.44, 0.74) | **0.3480**  0.498  0.090  0.789  0.623 | -2.01 (-14.54, 12.36)  (Reference group)  -4.37 (-18.54, 12.27)  0.96 (-14.37, 19.03)  -11.63 (-33.55, 17.52) | **0.8427**  0.771  0.585  0.909  0.395 | 0.04 (-0.11, 0.18)  (Reference group)  0.08 (-0.09, 0.25)  0.07 (-0.11, 0.24)  0.01 (-0.28, 0.29) | **0.8720**  0.622  0.362  0.453  0.969 | -0.03 (-0.09, 0.02)  (Reference group)  -0.03 (-0.08, 0.03)  0.02 (-0.04, 0.08)  -0.02 (-0.11, 0.06) | **0.0667**  0.263  0.397  0.495  0.608 |

1. **Univariate analysis of 16-haplotype classification and non-radiographic disease outcome measures in patients with RA.**

| *Haplotype* | **TJC** | | **SJC** | | **CRP** | | **DAS28** | | **HAQ** | |
| --- | --- | --- | --- | --- | --- | --- | --- | --- | --- | --- |
|  | *Coeff (95% CI)* | *p-value* | *Coeff (95% CI)* | *p-value* | *% change (95% CI)* | *p-value* | *Coeff (95% CI)* | *p-value* | *Coeff (95% CI)* | *p-value* |
| VKA | -0.06 (-0.36, 0.25) | 0.720 | 0.12 (-0.08, 0.33) | 0.247 | 14.15 (3.99, 25.30) | 0.005 | 0.03 (-0.07, 0.12) | 0.575 | -0.00 (-0.04, 0.03) | 0.805 |
| VRA | 0.09 (-0.27, 0.45) | 0.628 | 0.35 (0.09, 0.61) | 0.008 | 12.11 (-0.35, 26.13) | 0.057 | 0.16 (0.04, 0.29) | 0.011 | 0.01 (-0.03, 0.05) | 0.635 |
| LRA | -0.30 (-0.59, -0.00) | 0.048 | -0.23 (-0.45, -0.01) | 0.043 | 4.97 (-5.42, 16.51) | 0.362 | -0.09 (-0.19, 0.02) | 0.097 | 0.01 (-0.03, 0.05) | 0.500 |
| PAA | 0.16 (-0.17, 0.50) | 0.340 | -0.16 (-0.41, 0.10) | 0.233 | -17.10 (-25.95, -7.19) | 0.001 | -0.04 (-0.16, 0.08) | 0.492 | 0.01 (-0.04, 0.06) | 0.787 |
| GRQ | -0.09 (-0.44, 0.26) | 0.614 | -0.07 (-0.31, 0.16) | 0.532 | 2.80 (-7.50, 14.25) | 0.608 | 0.00 (-0.11, 0.12) | 0.934 | 0.05 (0.02, 0.09) | 0.004 |
| SRA | -0.00 (-0.46, 0.45) | 0.993 | -0.08 (-0.42, 0.27) | 0.660 | 2.94 (-11.77, 20.12) | 0.712 | -0.01 (-0.18, 0.16) | 0.917 | 0.00 (-0.07, 0.08) | 0.952 |
| SKR | 0.23 (-0.09, 0.56) | 0.154 | 0.24 (0.02, 0.46) | 0.034 | -3.05 (-12.38, 7.27) | 0.548 | 0.05 (-0.06, 0.15) | 0.414 | -0.00 (-0.04, 0.03) | 0.825 |
| SEA | 0.32 (-0.08, 0.72) | 0.116 | -0.09 (-0.37, 0.18) | 0.496 | -19.50 (-29.35, -8.29) | 0.001 | -0.05 (-0.18, 0.08) | 0.473 | -0.12 (-0.17, -0.08) | 4.46E-08 |

1. **Multivariate analysis of 16-haplotype classification and non-radiographic disease outcome measures in patients with RA.**

| *Haplotype* | **TJC** | | **SJC** | | **CRP** | | **DAS28** | | **HAQ** | |
| --- | --- | --- | --- | --- | --- | --- | --- | --- | --- | --- |
|  | *Coeff (95% CI)* | *p-value* | *Coeff (95% CI)* | *p-value* | *% change (95% CI)* | *p-value* | *Coeff (95% CI)* | *p-value* | *Coeff (95% CI)* | *p-value* |
| **Overall model** |  | **0.2837** |  | **0.0211** |  | **0.0001** |  | **0.1835** |  | **6.34E-06** |
| VKA | -0.17 (-0.59, 0.25) | 0.424 | 0.21 (-0.11, 0.52) | 0.197 | 30.77 (14.11, 49.87) | 1E-04 | 0.04 (-0.09, 0.18) | 0.535 | -0.01 (-0.07, 0.04) | 0.663 |
| VRA | -0.11 (-0.57, 0.36) | 0.659 | 0.45 (0.10, 0.80) | 0.011 | 28.78 (10.43, 50.18) | 0.001 | 0.17 (0.01, 0.33) | 0.038 | 0.03 (-0.03, 0.10) | 0.336 |
| LRA | -0.44 (-0.87, -0.01) | 0.045 | -0.06 (-0.38, 0.26) | 0.713 | 24.34 (7.23, 44.18) | 0.004 | -0.05 (-0.20, 0.10) | 0.550 | 0.00 (-0.05, 0.06) | 0.901 |
| PAA | (Reference group) |  | (Reference group) |  | (Reference group) |  | (Reference group) |  | (Reference group) |  |
| GRQ | -0.26 (-0.71, 0.19) | 0.265 | 0.07 (-0.26, 0.40) | 0.679 | 19.15 (3.11, 37.69) | 0.018 | 0.03 (-0.13, 0.19) | 0.706 | 0.06 (-0.00, 0.11) | 0.053 |
| SRA | -0.18 (-0.72, 0.35) | 0.500 | 0.07 (-0.33, 0.47) | 0.740 | 21.12 (1.20, 44.95) | 0.037 | 0.02 (-0.18, 0.21) | 0.866 | 0.02 (-0.06, 0.10) | 0.640 |
| SKR | 0.00 (-0.45, 0.45) | 0.994 | 0.35 (0.03, 0.67) | 0.034 | 16.00 (0.51, 33.89) | 0.042 | 0.08 (-0.08, 0.23) | 0.323 | 0.01 (-0.04, 0.07) | 0.619 |
| SEA | 0.12 (-0.39, 0.63) | 0.643 | 0.07 (-0.29, 0.44) | 0.689 | -3.14 (-17.90, 14.26) | 0.705 | -0.01 (-0.18, 0.16) | 0.876 | -0.09 (-0.16, -0.03) | 0.003 |

* *Remains significant following Benjamini-Hochberg correction for multiple testing.*

*Abbreviations*: NOAR = Norfolk Arthritis Register. AA = amino acid. RA = rheumatoid arthritis. TJC = tender joint count. SJC = swollen joint count. CRP = C-reaction protein. DAS28 = disease activity score of 28 joints. HAQ = Health Assessment Questionnaire. Coeff = β-coefficient – this represents an equivalent change in units of the variable of interest. 95% CI = 95% confidence intervals of β-coefficient value. Asp. acid = aspartic acid. Glut. acid = glutamic acid.

**Supplementary Table 4. Validation of IP disease outcome measures in ERAS cohort.**

| *AA/haplotype* | **TJC** | | **SJC** | | **ESR** | | **DAS28** | | **HAQ** | |
| --- | --- | --- | --- | --- | --- | --- | --- | --- | --- | --- |
|  | *Coeff (95% CI)* | *p-value* | *Coeff (95% CI)* | *p-value* | *% change (95% CI)* | *p-value* | *Coeff (95% CI)* | *p-value* | *Coeff (95% CI)* | *p-value* |
| Valine 11 | 0.09 (-0.19, 0.37) | 0.534 | 0.82 (0.47, 1.17) | 4.55E-06* | 5.60 (1.48, 9.91) | 0.007 | -0.00 (-0.07, 0.07) | 0.980 | 0.04 (0.01, 0.06) | 0.002* |
| Haplotype VKA | -0.52 (-0.97, -0.07) | 0.024 | 0.53 (0.04, 1.02) | 0.035 | 10.94 (5.51, 16.19) | 5E-05* | -0.15 (-0.26, -0.04) | 0.006 | 0.02 (-0.02, 0.05) | 0.421 |

Aspartic acid was excluded from multivariate analysis in the ERAS cohort due to a frequency of <10% in the population, to minimise skewing of results.

**Remains significant following Benjamini-Hochberg correction for multiple testing.*

*Abbreviations*: IP = inflammatory polyarthritis. ERAS = Early Rheumatoid Arthritis Study. AA = amino acid. TJC = tender joint count. SJC = swollen joint count. CRP = C-reaction protein. DAS28 = disease activity score of 28 joints. HAQ = Health Assessment Questionnaire. Coeff = β-coefficient – this represents an equivalent change in units of the variable of interest. 95% CI = 95% confidence intervals of β-coefficient value.

**Supplementary Table 5. Sub-analysis in NOAR patients with IP by ACPA status (seropositive and seronegative).**

| Variable | SJC | | CRP | |
| --- | --- | --- | --- | --- |
|  | Coefficient (95% CI) | p-value | % change (95% CI) | p-value |
| **Amino acids – univariate analysis** | | | | |
| *Valine 11*  All patients  ACPA seropositive patients  ACPA seronegative patients | 0.30 (0.17, 0.43)  0.18 (-0.06, 0.42)  0.18 (0.01, 0.35) | 7.51E-06  0.150  0.038 | 16.27 (9.23, 23.8)  3.58 (-5.83, 13.93)  -2.19 (-10.40, 6.77) | 2.21E-06  0.469  0.620 |
| *Serine 11*  All patients  ACPA seropositive patients  ACPA seronegative patients | -0.11 (-0.23, 0.01)  -0.06 (-0.33, 0.21)  0.01 (-0.13, 0.16) | 0.075  0.667  0.844 | -10.17 (-15.31, -4.72)  -0.71 (-9.88, 12.55)  0.80 (-6.06, 8.16) | 4E-04  0.900  0.825 |
| **Haplotypes – univariate analysis** | | | | |
| *VKA*  All patients  ACPA seropositive patients  ACPA seronegative patients | 0.29 (0.12, 0.46)  0.21 (-0.09, 0.51)  0.13 (-0.09, 0.35) | 0.001  0.165  0.262 | 16.00 (6.79, 26.00)  1.24 (-10.26, 14.22)  13.19 (-12.95, 47.18) | 4E-04  0.841  0.355 |
| *SEA*  All patients  ACPA seropositive patients  ACPA seronegative patients | -0.11 (-0.33, 0.12)  0.30 (-0.37, 0.97)  0.05 (-0.18, 0.28) | 0.351  0.379  0.683 | -17.81 (-25.67, -7.28)  -3.57 (-14.04, 8.18)  -8.26 (-18.58, 3.36) | 0.001  0.536  0.157 |

*Abbreviations*: NOAR = Norfolk Arthritis Register. IP = inflammatory polyarthritis. ACPA = anti-citrullinated protein antibody. SJC = swollen joint count. CRP = C-reaction protein. Coefficient - this represents an equivalent change in units of the variable of interest. 95% CI = 95% confidence intervals of β-coefficient value.

Only results for the most strongly positively and negatively associated amino acids/haplotypes are presented, from univariate analysis. There was no missing ACPA status for patients used for the analysis presented in this table (“All patients”).
